# Vegetation restoration effectiveness with main factors in the Beijing-Tianjin sandstorm source region during 2000–2020, China

**DOI:** 10.1371/journal.pone.0318176

**Published:** 2025-02-06

**Authors:** Xingshuo Zhang, Jingfang Yuan, Xiaoman Liu, Cheng Zong

**Affiliations:** 1 College of Wildlife and Protected Areas, Northeast Forestry University, Harbin, China; 2 Nanjing Institute of Environmental Sciences, Ministry of Ecology and Environment, Nanjing, China; 3 Satellite Application Center for Ecology and Environment, Ministry of Ecology and Environment, Beijing, China; Central South University of Forestry and Technology, CHINA

## Abstract

The Beijing-Tianjin Sandstorm Source Region (BTSSR), a region with significant vegetation degradation in China, has been subjected to ecological engineering intended to curb vegetation browning. Nevertheless, few studies have used multisource data to quantitatively evaluate the vegetation restoration effectiveness in the BTSSR, and the relationship between ecological engineering and vegetation restoration effectiveness in this region from statistical evidence has received little attention so far. Here, we employed the comprehensive vegetation parameters to describe the vegetation restoration effectiveness, and examined the driving mechanism of natural and human factors in different sub regions. First, we evaluated the vegetation restoration effectiveness in the BTSSR using an index that combined Fractional Vegetation Coverage (FVC) and Net Primary Productivity (NPP). Our results showed that the vegetation restoration effectiveness has significantly increased over time. From 2000 to 2020, 60.9% of the area achieved significant vegetation restoration, and the area with higher vegetation restoration effectiveness was concentrated in the southern part of the study area. Then, we used the Geodetector Model to explore the main factors and their interactions affecting vegetation restoration effectiveness. We found that the vegetation restoration effectiveness in the entire area was dominated by annual precipitation, in the northern part of the study area was led by climate, and in the southern part of the study area was dominated by ecological engineering. We further demonstrated that the interaction between ecological engineering and climate, soil conditions, geographical background and socioeconomic had the synergistic effect on vegetation restoration effectiveness, and the interaction between ecological engineering and annual precipitation had the greatest impact. We recommend that the northern region of the BTSSR continue to build low-density wind and sand control forests, while the southern region needs to be strengthened to prevent soil erosion problems caused by the expansion of human activities.

## Introduction

The large-scale climate change and rapid global socio-economic development pose threats to the health of natural resources and the ecological environment [1]. Scientists and decision-makers are increasingly turning to ecological restoration to address the issue of ecological degradation [2]. The Beijing-Tianjin Sandstorm Source Area, located in the ecologically fragile zone of northern China, has experienced severe land desertification, soil erosion, and vegetation degradation due to the continuous grassland cultivation and nomadic development. This posed a threat to the ecological security of Beijing and its surrounding areas [3]. In 2000, the Chinese government initiated the Beijing-Tianjin Sandstorm Source Control Project with the aim of controlling vegetation degradation, suppressing land desertification, and establishing a green ecological barrier in the Beijing-Tianjin Sandstorm Source Region (BTSSR) [4].

Now, after decades, it is imperative to evaluate the ecological project and adjust the implementation plan to ensure the effectiveness of ecological engineering. Ecological restoration typically relies on vegetation restoration, and the goal of ecological restoration is to enhance vegetation activity and enrich the ecosystem service value provided by vegetation. Therefore, vegetation restoration is commonly used to assess the success or failure of ecological restoration projects [5–6]. Vegetation constitutes the primary component of terrestrial ecosystems and plays a crucial role in preventing desertification, maintaining biodiversity, and conserving soil and water [7]. The dynamic changes of vegetation not only represent the characteristics of terrestrial ecosystems but also serve as important indicators of ecological environment change [8]. Since the implementation of the Beijing-Tianjin Sandstorm Source Control Project in 2000, it has mainly effectively suppressed sandstorms by improving vegetation and increasing surface roughness, preventing the exposed surface from being weathered and eroded to form new sand dunes, and slowing down the diffusion of existing sand dunes [9]. Vegetation restoration was the primary goal of the Beijing-Tianjin Sandstorm Source Control Project, and it was also an important criterion for judging the success of the project [10]. Therefore, long-term evaluation of the vegetation restoration effectiveness in the project can provide a scientific basis for adopting reasonable restoration strategies.

In monitoring large-scale vegetation dynamics and ecological restoration studies, remote sensing parameters are widely used because of their wide coverage and high temporal resolution [11]. Vegetation parameters, as the essential functional components of terrestrial ecosystems, encompass abundant surface structure and functional information. Previous studies have also utilized remote sensing vegetation indices to investigate vegetation dynamics in the BTSSR [4,10]. However, these studies often rely on a single indicator to assess the effectiveness of vegetation restoration, lacking comprehensive evaluation indicators capable of characterizing the temporal changes in restoration effectiveness [12]. Fraction Vegetation Coverage (FVC) and Net Primary Productivity (NPP) are two important parameters that reflect the ecological quality of vegetation. FVC is a significant parameter that characterizes vegetation cover structure and growth dynamics of vegetation communities [13], while NPP is a key indicator that reflects the production capacity of vegetation communities and their ecosystem functional characteristics [14]. The combination of these two indicators can provide insights into the vegetation restoration effectiveness by considering both vegetation structure and functional characteristics, thus addressing the limitation of relying on single indicator in vegetation restoration assessment.

Understanding the attribution analysis of large-scale and long-term vegetation changes is crucial for ecological restoration effectiveness research, which is a common demand in both academic research and management practice [11,15,16]. Previous studies frequently employed methods such as the correlation analysis [17], the residual analysis [18], and the multiple linear regression [19] to analyze the causes of vegetation change. However, all these methods assume a significant linear relationship between vegetation change and various influencing factors. Due to the strong interaction between natural and human factors, vegetation change exhibits significant spatial heterogeneity, suggesting that a strict linear relationship may not exist. Geodetector Model is a recent statistical method used to detect and reveal the driving mechanisms of spatial heterogeneity [20]. This model does not rely on linear assumptions or consider collinearity between variables. Its theoretical core is to detect the consistency of distribution patterns between dependent and independent variables by utilizing spatial heterogeneity, thereby revealing the underlying driving forces. It is a valuable approach to studying the influencing factors of vegetation restoration effectiveness using Geodetector Model [20].

Therefore, this study took the BTSSR as the study area, established a evaluation method suitable for vegetation restoration effectiveness, analyzed the vegetation restoration effectiveness of the BTSSR from 2000 to 2020, and revealed the explanatory power of different driving factors on vegetation restoration effectiveness by Geodetector Model. Our research aims to address the following scientific questions: (1) How effective has the BTSSR been in restoring vegetation over the past 20 years? (2) What are the primary factors influencing dynamic vegetation restoration? (3) Are climate factors or ecological engineering more significant in contributing to the vegetation restoration effectiveness? The findings will have a positive impact on future regional ecological restoration policies and other related areas.

## Materials and methods

### Study area information

**Fig 1:**
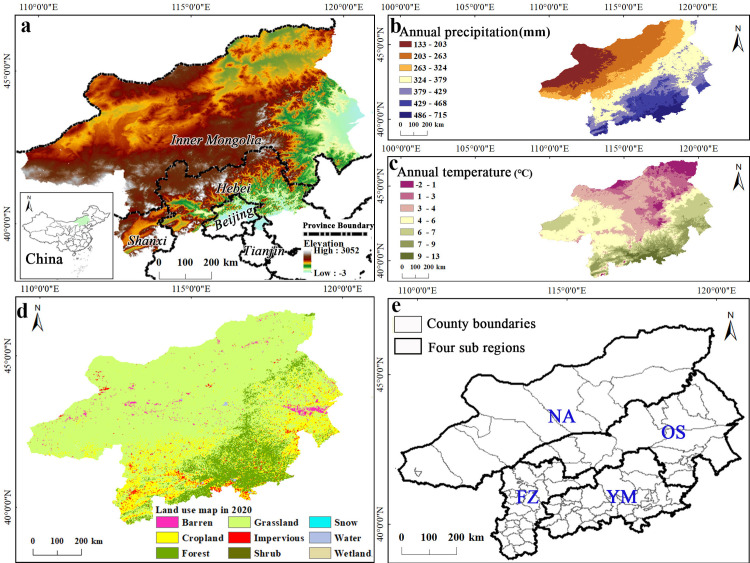
Background information of the study area. (a) Location of the study area; (b) and (c) are the annual average precipitation and the annual temperature from 2000 to 2020; (d) land use types in 2020; (e) four sub regions. The maps are based on the standard map with review number GS(2019)1822 downloaded from the Ministry of Natural Resources Standard Map Service website, with no modifications to the base map.

The Beijing-Tianjin Sandstorm Source Region (BTSSR) is an ecological engineering project area divided by China to improve the ecological environment of the capital and surrounding areas. The project, launched in 2001, aims to reduce soil erosion and sandstorms caused by the destruction of vegetation. The project implements comprehensive management strategies based on the vegetation protection, afforestation project, and grain for green. According to China’s State Forestry and Grassland Administration, during 2001 to 2014, the annual investment in the project exceeded CNY 1 billion, with a cumulative investment of more than CNY 31.6 billion Through these efforts, more than 5.11 million acres have been afforested, and 2.06 million acres of grassland have been preserved [21]. By the end of 2020, the BTSSR included 81 counties in Beijing, Tianjin, Hebei, Shanxi and Inner Mongolia, with a total area of 458,000 km^2^. The terrain is high in the west and low in the east (Fig 1a). The disparate landform types create different climates, soil and vegetation zones. The study area has the complex climate with spatial heterogeneity in temperature and precipitation. The annual temperature in Abaga Banner is 0.6 °C and that in Beijing reaches 12 °C, and the annual average precipitation decreases from south to north (Fig 1b and c). Grassland makes up the majority of the ecosystem type (58.1% of the area), with significantly more artificial vegetation in the east than in the west (Fig 1d); both vegetation quality and area show a decreasing trend from east to the west and from south to north, which is consistent with the distribution of precipitation [22]. The distribution of resources and the economic growth in the BTSSR are both out of balance. In the southern part of the study area, the population is large, and the economy is developed, but the intensive development of natural resources has resulted in many ecological spaces being crowded out. Economic growth is rather slow in the eastern and northern parts, and improper production and management have resulted in environmental degradation characterized by desertification.

BTSSR exhibits significant variations in natural and human activities across different ecological areas, as well as ecological restoration priorities. In order to scientifically evaluate different damaged areas, the research area was divided into four sub regions: Northern arid grassland desertification control area (NA), Otindag sandy land control area (OS), Farming pastoral zone desertification control area (FZ), Yanshan mountain water source protection area (YM) (Fig 1e and Table 1), with the following zoning principles: (1)Considering natural geographical features such as climate, geology, geomorphology, hydrology, vegetation, and soil; (2)Considering the similarity between damage characteristics and governance measures; (3)When dividing ecological sub areas, the integrity of administrative units was maintained, taking into account that the boundary of project implementation is the county (flag, city, district) administrative unit [23].

**Table 1:** Basic characteristics of four sub regions.

Sub regions	Area	Counties	Climate	Major problems
NA	17560 km^2^	10	Arid and semi-arid region, annual temperature 0 – 3.5 °C, annual precipitation 150 – 250 mm	Severe desertification and degradation of grasslands; The largest number of ecological refugees
OS	15430 km^2^	17	Semi-arid region, annual temperature 1.6 – 7.0°C, annual precipitation 300 – 400 mm	Grassland degradation; Rapid increase in quicksand area
FZ	5445 km^2^	24	Arid and semi-arid region, annual temperature 2 – 8°C, annual precipitation 250 – 450 mm	The soil is loose, with small sediment particles, making it prone to sand and dust
YM	73820 km^2^	30	Semi-arid and semi humid region, annual temperature 4 °C, annual precipitation 500 – 600 mm	Severe soil erosion and desertification

### Dataset and processing

The datasets used in this research for calculating vegetation restoration effectiveness and analysis of influencing factors are summarized in Table 2. Every remote sensing data was converted into the UTM coordinate system for projection purposes, and the maximum spatial resolution of 1000 m is sufficient to reflect spatial information due to the large spatial scale of the research area.

**Table 2:** Information of the datasets used for BTSSR.

Dataset	Temporal duration	Spatial resolution	Temporal resolution	Source
NPP	2000–2020	500 m	Yearly	MOD17A3HGF (https://Ipdaac.usgs.gov)
NDVI	2000–2020	500 m	8 days	MODIS spatial product dataset (http://ladsweb.nascom.nasa.gov)
Climate	2000–2020	1000 m	Yearly	1 km monthly temperature and precipitation dataset for China from 1901 to 2017 (http://www.geodata.cn)
Soil	1995	1000 m	–	HWSD (V1.2) (http://www.geodata.cn)
Digital Elevation Model (DEM)	2000	30 m	–	Shuttle Radar Topography Mission (https://www.resdc.cn)
GDP	2000, 2005, 2010, 2015, 2020	1000 m	Yearly	China GDP Spatial Distribution Kilometer Grid Dataset (https://www.resdc.cn)
Population density	2000, 2005, 2010, 2015, 2020	1000 m	Yearly	China Population Spatial Distribution Kilometer Grid Dataset (https://www.resdc.cn)
Land use data	2000 and 2020	1000 m	Yearly	Wuhan University CLCD Dataset (http://doi.org/10.5281/zenodo.8176941)
Afforestation data	2002–2020	County level data	Yearly	China Forestry and Grassland Yearbook

### Yearly FVC and NPP

FVC is the percentage value of green foliage coverage per unit land surface determined by vertical projection, which depicts the intuitive measurement of surface vegetation coverage and reflects the structure of vegetation [24]. NPP, which is the net amount of organic matter accumulated by plant photosynthesis minus the consumption of autotrophic respiration, is frequently used to indicate the function of vegetation [25]. FVC and NPP could illustrate how vegetation responds to natural and anthropogenic disturbances since they are sensitive to climate change and human activities [26]. Therefore, this study used FVC and NPP as datasets reflecting vegetation structure and function.

The FVC from 2000 to 2020 was available by calculating the Normalized Difference Vegetation Index (NDVI) through the dimidiate pixel model [27]. The NPP from 2000 to 2020 was directly derived from MOD17A3HGF product, which have been further improved the estimation accuracy of NPP and applied in different regions across the country [28–30]. After concatenating, cropping, and resampling the FVC and NPP data, annual FVC and NPP data covering the entire BTSSR from 2000 to 2020 were obtained, with a resolution of 1 km.

### Influencing factors

BTSSR is affected by various aspects of climate, soil, geography, socio-economic and ecological engineering. The spatial variation of temperature and precipitation in the BTSSR determines the distribution of water and heat, which directly impacts vegetation restoration [12]. The diverse topography of the BTSSR exhibits significant variations, with altitude playing a key role in the vertical distribution of vegetation and thereby controlling its growth [12]. Soil conditions determine water retention and ventilation, which are essential for regulating vegetation patterns in arid and semi-arid areas [31]. Furthermore, differences in socioeconomic conditions and governance priorities among sub regions lead to varying approaches to restoration measures [22]. As a result, the effectiveness of ecological engineering initiatives in the BTSSR and its sub regions also varies.

Given the above, this study collected five categories of climate, soil conditions, geo-graphical background, socioeconomic, and ecological engineering, for a total of 14 factors (Table 3). In the construction of plants, the Beijing-Tianjin Sandstorm Source Control Project involves reforestation, grassland management, and converting farmland back to forest (grassland). Therefore, we used the land use data in 2000 and 2020 to calculate the intensity of returning farmland to grass, the intensity of returning unused land to grassland, and the intensity of protecting the original forest (grassland) [32–33] and gathered county-level statistical data on afforestation during 2002 to 2020 (county-level afforestation statistics in 2000 and 2001 were not available), representing the construction of forest and grass in ecological engineering from five aspects. To spatialize ecological engineering data, the ecological engineering factors were normalized by the county area. This normalization allowed for the representation of ecological engineering on a per-unit area basis, facilitating the integration of statistical and spatial data.

**Table 3:** Factors affecting vegetation restoration effectiveness in BTSSR.

Categories	Factors	Interpretation and processing
Climate	Annual precipitation (mm)	Annual precipitation from 2000 to 2020, extracted from Climate dataset
	Annual temperature (°C)	Annual temperature from 2000 to 2020, extracted from Climate dataset
Soil conditions	Soil clay content (%)	Extracted from the Soil dataset
	Soil moisture content (mm/m)	
	Soil organic carbon content (%)	
Geographical background	Altitude (m)	Extracted from the DEM dataset
	Slope (°)	Calculated from DEM in ArcGIS 10.8
Socioeconomic	GDP (ten thousand yuan/ km^2^)	Average GDP for the five years 2000, 2005, 2010, 2015 and 2019, calculated from GDP dataset
	Population density (persons/km^2^)	Average population density for the five years 2000, 2005, 2010, 2015 and 2019, calculated from Population density dataset
Ecological engineering	Intensity of returning farmland to grassland (%)	The area ratio of land converted into grassland compared to the prior land, calculated from land use data
	Intensity of returning unused land to grassland (%)	
	Intensity of maintaining original forest (%)	The area ratio of unconverted forest or grassland compared to the prior land, calculated from land use data
		Intensity of maintaining original grassland (%)
	Accumulated afforestation intensity (%)	The area ratio of the accumulative afforestation area to the area of the county from 2002 to 2020

Before attribution analysis, the data for each factor must be discretized, and we must classify the factors in this study. The natural breaks method can achieve optimal grouping by minimizing the average deviation within each group while maximizing the average deviation of each group from the other groups. According to the natural breaks method, the climatic conditions (annual precipitation, annual average temperature), geological background (altitude, slope), and socioeconomic factors (GDP, population density) were divided into 7 groups, and the ecological engineering factors (intensity of returning farm-land to grassland, intensity of returning unused land to grassland, intensity of protecting original forest, intensity of protecting original grassland, accumulated afforestation intensity) were divided into 6 groups [34]. According to the Harmonized World Soil Database and BTSSR actual conditions, the soil clay content, soil moisture content, and soil organic carbon content were divided into 7, 6, and 5 groups, respectively.

## Methods

### Vegetation restoration effectiveness

This study constructed the vegetation restoration effectiveness index by integrating NPP and FVC, which reflect the structure and function of vegetation, and by setting a “baseline” to assess the changes in vegetation restoration effectiveness periodically [35–36]. The first phase of the Beijing-Tianjin Sandstorm Source Control Project ended in 2010, thus we used the average value of the vegetation restoration effectiveness from 2000 to 2010 as the “baseline” to calculate the vegetation restoration effectiveness from 2000 to 2010, 2000 to 2015, and 2000 to 2020 [12]. The specific methods are as follows:

(1) Vegetation Quality-Quantity Index (VQQI)

To combine the two parameters, the FVC and NPP were adjusted using normalization. Normalization is effective in both eliminating dimensional differences between parameters and retaining information about differences within parameters [37]. FVC represents vegetation coverage and plant structure, while NPP reflects vegetation productivity and measures vegetation function [38–39]. Considering that vegetation structure and function are equally important [40], FVC and NPP are given equal weights.


$$\begin{array}{c} VQQI=\left(N_{\text{FVC}}+N_{\text{NPP}}\right)/2\times 100\% \end{array}$$ (1)

VQQI means the vegetation quality-quantity index, distributed from 0–100%, *N*_FVC_ and *N*_NPP_ are FVC and NPP after normalization.

(2) Net change in vegetation in each county (Nt)

The spatial variation trend (Slope_*t*_) of the VQQI for each time period is calculated using the univariate linear regression approach [41]. The formula is as followed:


$$\begin{array}{c} {\text{Slope}}_{t}=\frac{l\sum_{n=2000}^{l}\left(n\times \text{VQQI}\right)-\sum_{n=2000}^{l}n\times \sum_{n=2000}^{l}\text{VQQI}}{l\sum_{n=2000}^{l}n^{2}-{\left(\sum_{n=2000}^{l}n\right)}^{2}} \end{array}$$ (2)

When Slope_*t*_ is positive, VQQI is rising, and negative means VQQI is falling, *t* represents different time scales (*t* = 2000–2010, 2000–2015, 2000–2020), *l* is the length of the time scale, and *n* represents different years (*n* = 2000, 2001...2020).

An F test was used to test the significance of Slope_*t*_ [42]. According to the test results, Slope_*t*_ was divided into 5 grades, and based on previous studies, weights were assigned to Slope_*t*_ of different grades [12]: extremely significant improvement (Slope_*t*_ > 0, *p* < 0.01), significantly improved (Slopet > 0, 0.01 <  *p* < 0.05), not significantly changed (*p* > 0.05), significantly degraded (Slopet < 0, 0.01 < *p* < 0.05), and extremely significantly degraded (Slopet < 0, *p* < 0.01) were assigned weights 2, 1, 0, −1, −2, respectively. Taking each county of the BTSSR as the evaluated unit, the net change in vegetation restoration (N_*t*_) can be obtained by the following formula:


$$\begin{array}{c} N_{t}=\sum_{k=1}^{5}(\omega_{k}\times A_{k,t}) \end{array}$$ (3)

A_*k,t*_ represents the area ratio of different grades in the evaluated county under each time scale, *k* represents different grades (*k* = 1, 2,..., 5), and ω_*k*_ represents the weight of different grades.

(3) Vegetation restoration effectiveness (*E*_*t*_)

The vegetation restoration effectiveness (*E*_*t*_) in each period is calculated using the “baseline” ($\bar{N_{0}}$), which is the average value of the vegetation restoration effectiveness from 2000 to 2010.


$$\begin{array}{c} \bar{N_{0}}={\left[\sum_{k=1}^{5}(\omega_{k}\times A_{k,0})\right]}_{avg} \end{array}$$ (4)

$$\begin{array}{c} E_{t}=N_{t}/\bar{N_{0}} \end{array}$$ (5)

The formulas above can be used to determine how the vegetation restoration effectiveness index (*E*_*t*_) of BTSSR counties changes over time. When *E*_*t*_ ≤ 0, the majority of the county’s area has experienced significant vegetation degradation, and the county has experienced vegetation degradation during this time. When 0 < *E*_*t*_ ≤ 1, it indicates that the county’s vegetation has recovered but has not yet reached the “baseline” and has not recovered significantly. When 1 <  *E*_*t*_, it indicates that the county’s vegetation has significantly recovered during this time.

### Geodetector model to quantify the contribution of major factors

By statistically identifying the connection between variables, the Geodetector Model is a spatial statistical tool that evaluates the contribution of the main factors to the response variable [43]. First, we used Geodetector Model to quantitatively filter the main factors that largely affected the vegetation restoration effectiveness in the BTSSR, this study selected the factors whose *q* value ranks in the top 50% and passes the 95% confidence level as the main factors; then, we determined the contribution of every single main factor to the vegetation restoration effectiveness and the optimal range of each main factor for vegetation restoration in the four sub regions (NA, OS, FZ and YM); finally, we used Geodetector Model to assess the two-by-two interactions between the main factors on the vegetation restoration effectiveness in each ecological area. The contribution of single main factors and the interaction were measured by the *q* value.


$$\begin{array}{c} q=1-\frac{\sum_{h=1}^{L}N_{h}\sigma_{h}{}^{2}}{N\sigma^{2}} \end{array}$$ (6)

The range of the *q* value is [0,1], and with the increase in *q* values, the explanatory power of factor is expected to be stronger. According to the factor or the vegetation restoration effectiveness, we divide the study area into *h* (*h* = 1, 2..., L) layers; *N*_*h*_ and *N* represent the number of units in *h* and the entire area, respectively; and σ_*h*_^2^ and σ^2^ are the variance in vegetation restoration effectiveness in *h* and the entire area, respectively.

## Results

### VQQI changes in BTSSR from 2000 to 2020

**Fig 2:**
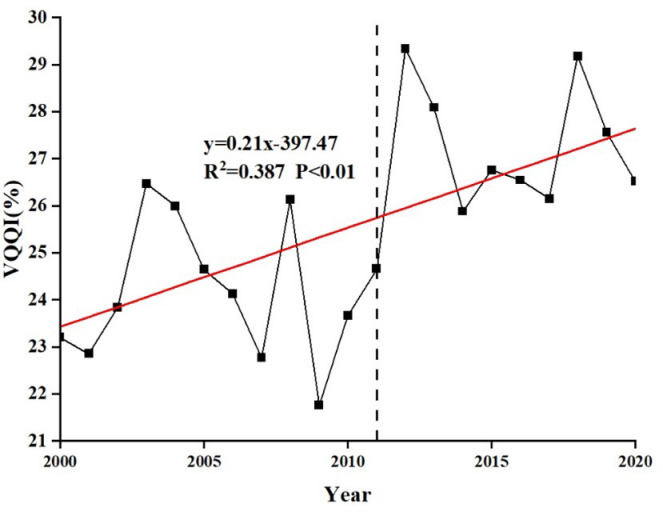
The annual change trend of the VQQI during 2000–2020 in BTSSR.

From 2000 to 2020, the annual average VQQI in BTSSR exhibited the increasing trend, with a growth rate of 0.21% per year (*p* < 0.01), which suggested a positive trend in vegetation growth and an overall enhancement in vegetation quality (Fig 2). Throughout the study period, the average VQQI was 26%, with a notable 14.3% increase in 2020 compared to 2000. According to mutation analysis, the year of mutation for the average vegetation index in BTSSR was 2011. From 2000 to 2010, VQQI ranged from 21% to 27%, averaging at 24%. Subsequently, from 2011 to 2020, VQQI ranged from 24% to 30%, with a mean of 27% (Fig 2). Nevertheless, VQQI in this region exhibited significant fluctuations, particularly in 2007 and 2009, coinciding with sharp declines. These declines were attributed to extreme drought conditions during the summers of 2007 and 2009, leading to a substantial decrease in VQQI during those years [44].

### Vegetation restoration effectiveness on different time scales

**Fig 3:**
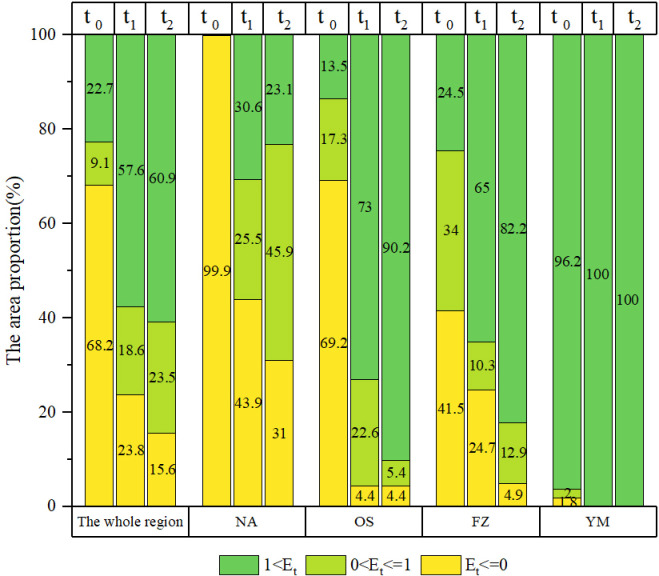
Area proportion of vegetation restoration effectiveness in BTSSR and its sub regions on different time scales, t_0_ means 2000–2010, t_1_ means 2000–2015, t_2_ means 2000–2020.

**Fig 4:**
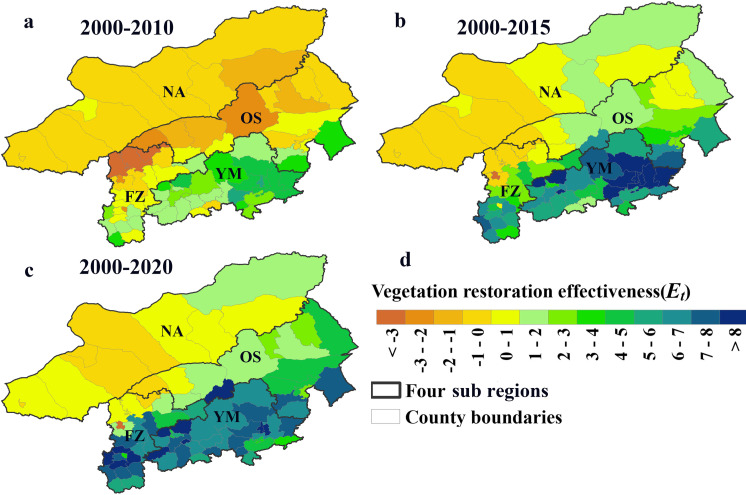
Distribution map of vegetation restoration effectiveness in BTSSR and its sub regions on different time scales (a: 2000–2010; b: 2000–2015; c: 2000–2020; d: legend). The maps are based on the standard map with review number GS(2019)1822 downloaded from the Ministry of Natural Resources Standard Map Service website, with no modifications to the base map.

The vegetation restoration effectiveness shown in Figs 3 and 4 indicated that since 2000, the vegetation restoration effectiveness of different grades in various BTSSR areas has varied greatly throughout the three time scales: (1) in the early stage of vegetation restoration (2000–2010), the vegetation degradation area accounted for the main part of the study area (68.2% of the area, including 28 counties), the area ratio of the significant vegetation restoration land to the whole land reached 22.70%, and these counties were mainly located in YM (Figs 3 and 4a); (2) with the extension of the time scale (2000–2015), the area of counties with significant vegetation restoration made up 57.62% of the total study area and had expanded by 1.6 times since the previous stage. However, compared to the prior scale, there was an increase in the area of nonsignificant vegetation restoration (Figs 3 and 4b); (3) On a longer time scale (2000–2020), the area of significant vegetation restoration reached its peak at 60.9%, and the regions with higher vegetation restoration effectiveness values were mainly distributed in the south of the study area, and the land use types were mainly forest and cropland (Figs 4c and 1d). In the period from 2000 to 2020, 66 counties in the BTSSR, accounting for 60.9% of the total area, achieved significant vegetation restoration. According to the proportion of significantly restored vegetation area, the ecological areas were ranked in decreasing order as follows: YM (100.0%) > OS (90.2%) > FZ (82.2%) > NA (23.1%). It was NA, where 88.7% of the vegetation degraded area in the BTSSR came from, reducing the vegetation restoration effectiveness in the entire study area.

### Contribution of the main factors to vegetation restoration effectiveness

To filter the main factors affecting the vegetation restoration effectiveness in the BTSSR, the *q* value was used to rank the contribution of each factor in the whole region: annual precipitation (0.75) > GDP (0.61) > accumulated afforestation intensity (0.57) = intensity of returning farmland to grassland (0.57) > population density (0.56) > annual temperature (0.42) > slope (0.27) > intensity of returning unused land to grassland (0.26) > intensity of maintaining original grassland (0.22) > altitude (0.20) > intensity of maintaining original forest (0.19) > soil organic carbon content (0.05) > soil clay content (0.04) > soil moisture content (0.03). Finally, annual precipitation and annual temperature represented the climate; slope represented geographical background; GDP and population density represented the social economy; and accumulated afforestation intensity and intensity of returning farmland to grassland represented ecological engineering.

Since the natural surroundings and human activities in the four sub regions had evident geographical differences, it was necessary for more accurate governance to explore the driving conditions of various factors in different ecological areas. In different sub regions, the impacts of the factors on the vegetation restoration effectiveness were quite different. As shown in Table 4, the climatic conditions on NA (*q*(annual precipitation) = 0.40, *q*(annual temperature) = 0.56), OS (*q*(annual precipitation) = 0.56, *q*(annual temperature) = 0.31), and FZ (*q*(annual precipitation) = 0.45, *q*(annual temperature) = 0.31) had the higher effect and had no significant effect on YM (p > 0.05). Similarly, the *q* value reflecting geography was the highest in OS (*q*(slope) = 0.24) but had no significant effect on YM (*p* > 0.05). Regional consistency existed in the impacts of GDP and human density on vegetation restoration effectiveness; human activities had the greatest impact on the vegetation restoration effectiveness in OS, whereas they had the least impact on the effect in NA. In locations with greater water and heat conditions, such as the YM (*q*(afforestation intensity) = 0.23) and FZ (*q*(returning farmland to grassland) = 0.52), ecological engineering had a significant impact on the vegetation restoration effectiveness.

**Table 4:** The q value in different sub regions.

Four sub rejoins	annual precipitation	annual temperature	slope	GDP	population density	accumulated afforestation	intensity of returning farmland to grassland
**NA**	0.40*	0.56*	0.20*	0.02*	0.01	0.02*	0.20*
**OS**	0.56*	0.31*	0.24*	0.45*	0.28*	0.44*	0.50*
**FZ**	0.45*	0.31*	0.16*	0.33*	0.13*	0.29*	0.52*
**YM**	0.07	0.05	0.04	0.15*	0.04	0.23*	0.01

“*” indicates that the q value passes the test at the 95% confidence level, and orange indicates that this factor is the strongest driving factor in the ecological area.

**Fig 5:**
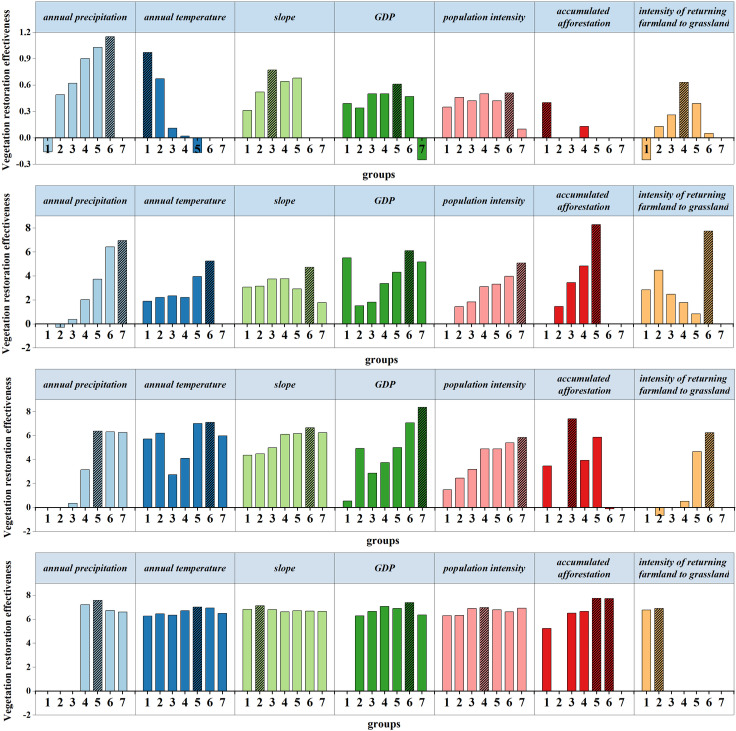
Distribution characteristics of vegetation restoration effectiveness on the main factor groups in the four ecological areas. (a) NA; (b) OS; (c) FZ; (d) YM.

Here we further examined the range of the strongest driving factors that encouraged vegetation growth in each sub region (Fig 5). To determine the range of the strongest driving factors for promoting vegetation growth in each sub region, we used Geodetector Model to detect the distribution characteristics of vegetation restoration effectiveness in different groups of the strongest driving factors. As a result, the higher the vegetation restoration effectiveness was, the more suitable each driving factor was for vegetation growth (Table 4). In the NA, temperature limited vegetation growth because the vegetation restoration effectiveness dropped as the annual temperature increased, and the vegetation restoration effectiveness was at its maximum when the annual temperature was at its lowest (–2.18 − 1.65 °C). In OS, the vegetation restoration effectiveness increased as the annual precipitation increased, peaking when the annual precipitation was between 486.83 and 715.01 mm. In FZ, the vegetation restoration effectiveness grew as the intensity of returning farmland to grassland increased. Although the vegetation restoration effectiveness reached a maximum (6.24) in Group 6 in returning farmland to grassland, it increased 8 times from Group 4 to Group 5 and only 1.3 times from Group 5 to Group 6. In YM, the vegetation restoration effectiveness increased with the growth of accumulated afforestation intensity, but there was no obvious difference in the vegetation restoration effectiveness between Group 5 and Group 6 in accumulated afforestation intensity. These two groups functioned similarly to plants, so the vegetation restoration effectiveness peaked at 0.24–1% (the range included Group 5 and Group 6) accumulated afforestation intensity.

With the group number increased, the factor value increased.

### Interaction between ecological engineering and other factors

**Fig 6:**
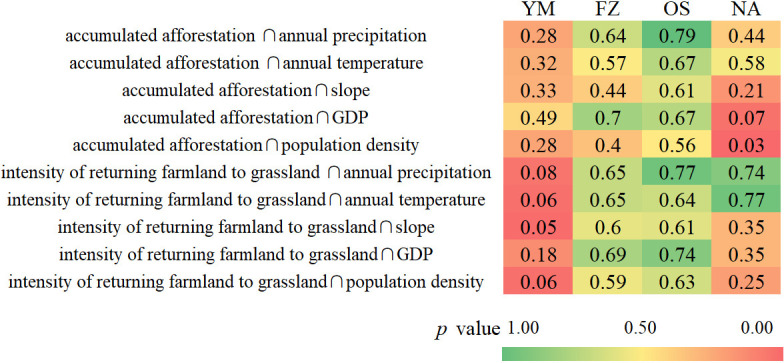
Interaction between ecological engineering and other factors in four sub regions. The interaction is indicated by “ ∩ ”.

The impacts of ecological engineering and other factors on the vegetation restoration effectiveness were not independent. As a result, we undertook interactive detection of ecological engineering factors combined with other factors to assess the impact of the natural and social factors on the ecological engineering effect. As shown in Fig 6, the research showed that the combination of ecological engineering and natural factors could enhance the vegetation restoration effectiveness, and the spatial heterogeneity of vegetation restoration effectiveness was not controlled by a single factor. Among all the interactions, the interaction between the intensity of returning farmland to grassland and annual precipitation had the largest impact on the vegetation restoration effectiveness (average *q* = 0.56), followed by the interaction between the accumulated afforestation intensity and annual precipitation (average *q* = 0.54). The most effective interaction in both OS (*q* = 0.89) and FZ (*q* = 0.79) was the interaction between ecological engineering; the most influential interaction in NA (*q* = 0.79) was between the intensity of returning farmland to grassland and the average annual temperature; in YM, the interaction between the accumulated afforestation intensity and GDP had the largest response to the vegetation restoration effective (*q* = 0.48).

## Discussion

### Vegetation restoration effectiveness in the BTSSR

This study used an evaluation method for assessing the vegetation restoration effectiveness at different stages, considering both vegetation structure and function. In contrast to previous studies that rely on a single parameter to assess vegetation recovery in the BTSSR [10,45,46], this method overcomes the limitation of relying on a single vegetation recovery indicator.

Previous studies have shown that the vegetation in BSSTR has been dominated by restoration over the past 20 years [45,47]. This study also obtained similar results: the area of significant vegetation restoration increased over time; from the early stage (2000–2010) to the middle stage (2000–2015), the area of significant vegetation restoration increased by nearly tripled, ultimately reaching a peak of 60.9% in the final phase (2000–2020). Geographically, the area of significant vegetation restoration has transitioned from an initial sporadic distribution to a more uniform distribution as the restoration progresses. During the early stage, the vegetation restoration effectiveness in most regions was degraded. However, the 2010 China Grassland Monitoring Report [48] showed that compared with the non-study area, the average vegetation coverage in the BTSSR increased by 15%, and the vegetation height increased by 54.1%. In addition to vegetation structure, the vegetation community has evolved from a single shrub vegetation to a composite vegetation system of trees, shrubs and grasses, and after grazing exclusion, the number of plant species in heavily degraded grassland communities increased from 7 to 11 species. The restoration of vegetation in the early stage (2000–2010) was mainly represented by the succession of vegetation communities to higher-level communities, which laid a foundation for the significant improvement of vegetation restoration in the later stage.

From 2000 to 2020, the counties with high vegetation restoration effectiveness were mainly concentrated in YM and south of FZ (Fig 4). Yu et al. [10] also proved that the vegetative background in this area was generally healthy, and vegetation has greatly recovered owing to a combination of climatic and human activities. Moreover, other studies have shown that vegetation in the southwest and east of the BTSSR has been degraded [46]. However, we found that the vegetation degradation counties were mainly located in the west of NA (Fig 4), where the area of degraded vegetation was larger than the area of restored vegetation. NA, located in arid and semi-arid regions, where water resources are relatively precious, might cause water deficits due to the vigorous growth of vegetation. Therefore, the measure for vegetation construction in NA was to make the density of windbreaks and sand fixation forests low (FVC is approximately 20%), which could save more than 50% of water resources compared to traditional random afforestation or block afforestation without lowering the impact of windbreaks and sand fixation [49]. Even though the NA’s western regions have experienced a relatively slow degree of vegetation restoration, the water resource guarantee capacity was improved when building windbreaks and sand fixation vegetation, and the sustainability of vegetation restoration was guaranteed.

### Dominant factors of vegetation restoration effectiveness

There are notable variations in the dominant factors influencing the vegetation restoration effectiveness among different sub regions in the BTSSR. NA and OS are mainly influenced by climate conditions, but the dominant factors are different. The vegetation restoration effectiveness in NA is primarily influenced by temperature (Table 4). An increase in temperature directly impacts the suitability of vegetation growth (Fig 5). Despite evidence supporting the idea that higher temperatures can extend the growth season and expedite plant recovery [50], this area is located at the arid climate zone. In such zones, elevated temperature led to increased evaporation of soil water, decreased surface soil water, and unfavorable conditions for the growth of shallow-rooted plants like grasses and cultivated crops, thereby impeding plant growth [51]. In OS, annual precipitation is the decisive factor in vegetation restoration (Table 4), and the vegetation restoration effectiveness improved with the increase of annual precipitation (Fig 5). The study area is located in a semi-arid climate zone, and the increase in precipitation aids in mitigating soil erosion, thus suppressing desertification and facilitating favorable conditions for vegetation restoration.

Ecological engineering was the dominant factor in the change in the vegetation restoration effectiveness in FZ and YM, where there have high annual precipitation and a more stable climate that are conducive to establishing forests and grass [52]. The intensity of returning farmland to grassland was the leading factor in the change in vegetation restoration effectiveness in FZ. FZ was a grassland sand region, with 33.2% of its total area being farmed, yet the soil in this area was loose. In addition to deforestation and grassland loss for reclamation and excessive grazing, the depth of wind erosion on the surface of cultivated land reached a decrease of 1–2 cm each year, which made the land unsuitable for farming, therefore, the policy of limiting reclamation was implemented [53]. After the policy, the measure of returning farmland to grassland achieved artificial greening on low-yield farmland, resulting in significant vegetation recovery. The accumulated afforestation intensity determined the vegetation restoration effectiveness in YM. This region’s climate is favorable for tree development since it has a constant temperature and 500–600 mm precipitation annually [54]. Consequently, the afforestation efforts in this area were relatively successful.

### Management suggestions for improving vegetation restoration effectiveness in different ecological restoration areas

Effective management strategies must adapt to local conditions and account for the comprehensive impacts of climate, soil, and human activities on different types of ecosystems. BTSSR is characterized by complex climate zones, variable topography, and different degrees of human activities. The different vegetation restoration effectiveness in different four ecological areas might be further explained by the interactions between ecological engineering and natural and human factors. Quantifying these interactions would help policy-makers to accurately restore regions according to different regional characteristics.

Low precipitation, high evapotranspiration, overgrazing by people, and mining development have all contributed to a weak vegetative base and a challenging recovery in the NA. For this fragile habitat area with low vegetation restoration effectiveness, it is necessary to strengthen grazing management to reduce grassland pressure, with ecological engineering focuses on improving grassland. In OS, the arid and semi-arid climate has restricted the growth of canopy, resulting in the succession of understory and inter-row vegetation still in the initial stage at the end of the first phase project [55]. Wei et al. [56] pointed out that extreme drought restrained the growth of grassland species such as Lespedeza, but had little impact on drought tolerant artificial forests such as Armeniaca sibirica and Artemisia annua. Therefore, the selection of artificial vegetation in OS should adapt to the semi-arid climate. In FZ, loose soil and windy days lead to desertification in the area, making plants difficult to grow, which explained why the combination of afforestation activities and agricultural management made the vegetation recovery in this area more effective. YM is located in hilly area, with high population density, large slope and rainfall, which is at the risk of soil erosion. With the vegetation restoring, more soil could be retained and more water would be retained in the soil, which in turn might continue to support vegetation growth [57]. Therefore, strengthening land use management and reducing human interference were effective ways to prevent soil erosion and vegetation degradation.

### Novelty and uncertainty

First, our study provides a framework integrating vegetation quality and vegetation coverage, which overcomes the problem of a single vegetation restoration index. Second, our framework can reflect the vegetation restoration in the county and provide a reference for researchers and managers for further research and planning. Third, we used GDM to quantify and distinguish the impacts of climate, geography, socioeconomic and ecological engineering factors on plant restoration in different ecological areas. Due to global climate change and intensified human activities, ecological engineering is being carried out globally to limit vegetation degradation. With the progress of ecological restoration, zoning policies and precise restoration have become important tasks of ecological governance. Our results can provide ideas for vegetation dynamic changes and driving mechanisms at work in northern Chinese ecological restoration zones.

We determined the vegetation restoration effectiveness in the BTSSR during the past 20 years and emphasized the effect of ecological engineering on vegetation protection. Nevertheless, the spatial and temporal resolution of the data in this study still has some limitations. Above all, the satellite images we used were moderate resolution. If we want to further understand the changes in species and the survival rate of vegetation, field surveys and higher resolution images for analysis may provide a more accurate perspective. Second, due to the limitation of data acquisition, this study did not consider the effects of grazing intensity, irrigated areas and water conservation efforts due to data gathering limitations. Unscientific planting might result in excessive water absorption and decreased water supplies in the desertification region [58]. Therefore, in arid and semi-arid environments, further in-depth studies are needed to understand the interplay between vegetation water absorption brought by ecological engineering and the water resource background.

## Conclusions

This study built a comprehensive index that reflected the effectiveness of vegetation restoration using FVC and NPP, and the study staged the 21-year BTSSR’s vegetation restoration effectiveness and examined the factors that influenced it. From the analysis above, the following conclusions can be drawn. The area of vegetation restoration has grown steadily since the start of the Beijing-Tianjin Sandstorm Source Project in 2000. As of 2020, the area with significant vegetation restoration accounted for 60.9%, and the high-value area for vegetation restoration was concentrated in YM and FZ; the area of vegetation degradation accounted for 15.59%, concentrated in the northwest part of NA. The vegetation restoration effectiveness in each ecological area of the BTSSR was affected by various factors, such as climate, geography, society and ecological engineering. In the YM and FZ, ecological engineering was the primary factor for vegetation restoration, while the vegetation restoration efficiency in the NA and OS was mostly determined by climatic conditions. Additionally, the impact of ecological engineering combined with other factors was stronger than the impact of a single factor on vegetation restoration. The interaction between ecological measures and annual precipitation is the most significant, suggesting that local climate, as well as geographical and social contexts, should be accounted for when planning and assessing ecological engineering.

## Supporting information

S1 Data Results in the vegetation restoration effectiveness and main factors. (XLSX)
